# Comparative genomics confirms a rare melioidosis human-to-human transmission event and reveals incorrect phylogenomic reconstruction due to polyclonality

**DOI:** 10.1099/mgen.0.000326

**Published:** 2020-01-20

**Authors:** Ammar Aziz, Bart J. Currie, Mark Mayo, Derek S. Sarovich, Erin P. Price

**Affiliations:** ^1^​ Global and Tropical Health Division, Menzies School of Health Research, Charles Darwin University, Darwin, NT, Australia; ^2^​ Infectious Diseases Department, Royal Darwin Hospital, Darwin, NT, Australia; ^3^​ GeneCology Research Centre, University of the Sunshine Coast, Sippy Downs, QLD, Australia; ^4^​ Sunshine Coast Health Institute, Birtinya, QLD, Australia

**Keywords:** human-to-human transmission, *Burkholderia pseudomallei*, phylogenomics, strain mixtures, phylogenetic incongruence, branch collapse

## Abstract

Human-to-human transmission of the melioidosis bacterium, *Burkholderia pseudomallei *, is exceedingly rare, with only a handful of suspected cases documented to date. Here, we used whole-genome sequencing (WGS) to characterize one such unusual *B. pseudomallei* transmission event, which occurred between a breastfeeding mother with mastitis and her child. Two strains corresponding to multilocus sequence types (STs)-259 and -261 were identified in the mother’s sputum from both the primary culture sweep and in purified colonies, confirming an unusual polyclonal infection in this patient. In contrast, primary culture sweeps of the mother’s breast milk and the child’s cerebrospinal fluid and blood samples contained only ST-259, indicating monoclonal transmission to the child. Analysis of purified ST-259 isolates showed no genetic variation between mother and baby isolates, providing the strongest possible evidence of *B. pseudomallei* human-to-human transmission, probably via breastfeeding. Next, phylogenomic analysis of all isolates, including the mother’s mixed ST-259/ST-261 sputum sample, was performed to investigate the effects of mixtures on phylogenetic inference. Inclusion of this mixture caused a dramatic reduction in the number of informative SNPs, resulting in branch collapse of ST-259 and ST-261 isolates, and several instances of incorrect topology in a global *B. pseudomallei* phylogeny, resulting in phylogenetic incongruence. Although phylogenomics can provide clues about the presence of mixtures within WGS datasets, our results demonstrate that this methodology can lead to phylogenetic misinterpretation if mixed genomes are not correctly identified and omitted. Using current bioinformatic tools, we demonstrate a robust method for bacterial mixture identification and strain parsing that avoids these pitfalls.

## Data Summary

1. Whole-genome sequencing data have been deposited in the NCBI Sequence Read Archive (SRA) and GenBank under BioProject accession number PRJNA559002.

2. The GenBank accession number for the MSHR0643 assembly is VXLH00000000.1.

3. The SRA accession numbers for all raw sequence data are listed in Table 1.

**Impact Statement**

*
Burkholderia pseudomallei* is the causative agent of melioidosis, a tropical disease of high mortality. *B. pseudomallei* infections occur almost exclusively through contact with contaminated soil and water; person-to-person transmission is uncommon. Using whole-genome sequencing (WGS), we investigated a rare case of suspected *B. pseudomallei* transmission from mother to child. The mother’s sputum, breast milk and the baby’s blood and cerebrospinal fluid specimens were collected, and DNA was extracted from both pure colonies and primary culture sweeps to capture potential strain mixtures. In-depth analysis of genetic variants identified two strains in the mother’s sputum belonging to multilocus sequence types (STs) ST-259 and ST-261, whereas the child was infected with only ST-259. Comparative genomics revealed no genetic differences between mother and child ST-259 isolates, providing the strongest possible evidence of transmission to the child via breast milk. The sputum strain mixture was subsequently used to develop a bioinformatic method for identification and quantification of mixtures from WGS data. Using this method, we found ST-259 and ST-261 present at an 87 %:13 % ratio, respectively. Finally, we demonstrate the negative impact that even a single strain mixture event can have on both within-ST and global phylogenomic inferences. Our findings highlight the need for bioinformatic quality control to avoid unintended consequences of phylogenomic incongruence and branch collapse caused by mixed genomes.

**Table 1.** Summary of ST-259 and ST-261 *B. pseudomallei* isolates

Isolate ID*	Sample type	Patient	Year of isolation	Multilocus ST	NCBI accession number	Genome coverage
MSHR1574	CSF	Child	2003	ST-259	SRR9959037	134×
MSHR1574_Sweep	CSF	Child	2003	ST-259	SRR9959038	94×
MSHR1580	Blood	Child	2003	ST-259	SRR9959039	109×
MSHR1580_Sweep	Blood	Child	2003	ST-259	SRR9959040	63×
MSHR1583	Breast milk	Mother	2003	ST-259	SRR9959042	125×
MSHR1583_Sweep	Breast milk	Mother	2003	ST-259	SRR9959036	68×
MSHR1631	Sputum	Mother	2003	ST-259	SRR9959045	131×
MSHR1631_Mixed	Sputum	Mother	2003	ST-259 and ST-261	SRR9959043	60×
MSHR1581	Sputum	Mother	2003	ST-261	SRR9959044	134×
MSHR1581_Sweep	Sputum	Mother	2003	ST-261	SRR9959041	61×
MSHR0120	Blood	Other†	1992	ST-259	SRR2975709	31×
MSHR0669	Blood	Other†	1998	ST-259	SRR9959034	111×
MSHR1224	Blood	Other†	2001	ST-259	SRR9959035	64×
MSHR1328	Sputum	Other†	2001	ST-259	SRR10134765	123×
MSHR1357	Abscess	Other†	2002	ST-259	SRR10134764	139×
MSHR3509	Blood	Other†	2009	ST-259	SRR10134763	97×
MSHR0643	Urine	Other†	1998	ST-259	SRR9959033	122×

*Isolates with the ‘_Sweep’ suffix were obtained from primary culture sweeps to capture *B. pseudomallei* population diversity. MSHR1631_Mixed was the only sample found to contain a mixture of two genotypes. Isolates without the ‘_Sweep’ suffix were obtained from purified single colonies derived from the ‘_Sweep’ culture.

†Temporally or geographically distinct clinical ST-259 isolates obtained between 1992 and 2009 from other patients living in the Top End region of the Northern Territory, Australia.

## Introduction

*Burkholderia pseudomallei *, a Gram-negative environmental bacterium found in soil and water in mostly tropical regions, is the causative agent of melioidosis [1]. This underreported and historically neglected disease is increasingly being recognized as endemic in diverse tropical regions globally, and is hyperendemic in northern Australia and Southeast Asia [2]. *B. pseudomallei* is an opportunistic bacterium that most commonly affects people who are in regular contact with soil and water, with percutaneous inoculation and inhalation the main routes of infection, and infection by ingestion uncommon [1, 3]. The high mortality rate of melioidosis (10–40 %) even with antibiotic treatment [4], combined with the intrinsic resistance of *B. pseudomallei* towards a wide range of antibiotics [5], highlight the significant public-health importance of this bacterium [1]. Increasing awareness and detection of melioidosis in new locales and the lack of a vaccine against *B. pseudomallei* have further increased the global public-health significance of this pathogen [6]. Due to these factors, *B. pseudomallei* is considered a Tier 1 Select Agent pathogen due to its potential for misuse as a biological warfare agent [7].

Multilocus sequence typing (MLST) is a commonly used genotyping method for determining the population structure, geography, source attribution and transmission patterns of many bacterial pathogens, including *B. pseudomallei* [8–14]. With the advent of whole-genome sequencing (WGS), simultaneous genomic characterization, phylogeography, multilocus sequence type (ST) determination, antibiotic-resistance profiling and fine-scale resolution of *B. pseudomallei* population structure, evolution and transmission profiles have become possible [15]. WGS has also assisted with the identification of polyclonal *B. pseudomallei* infections, including one reported instance of a polyclonal infection with the same ST [16].

Although rare, a handful of suspected cases of human-to-human *B. pseudomallei* transmission have been documented, including between siblings with cystic fibrosis [17], between siblings with diabetes [18], between an American Vietnam veteran diagnosed with *B. pseudomallei *-associated prostatitis and his spouse (although supported only by serology) [19], and three cases between mother and child [3, 20]. In one of the mother-to-child transmission cases, a mother with *B. pseudomallei *-associated mastitis in her left breast was suspected to have transmitted this pathogen to her breastfed infant [3]. Mother-to-child *B. pseudomallei* transmission via transplacental, breast or perinatal routes has been suspected in a handful of other human cases [3, 20, 21], and in animals [22]. However, no human-to-human transmissions reported to date have been confirmed using WGS, which is essential for ruling out concomitant environmental sources of infection. In the current study, WGS was used to understand the dynamics of this suspected human-to-human transmission event, which was also characterized by a polyclonal infection detected in the mother’s sputum. Using comparative genomics, we provide the strongest possible evidence for human-to-human *B. pseudomallei* transmission between mother and child. We next examined the impact of the strain mixture identified in the mother’s sputum sample on phylogenetic interpretations. We observed confounding phylogenomic results when the single mixed genome was included in analyses of both single-ST and highly diverse global strain phylogenies, a finding that has implications for fine-scale phylogenomic investigation of outbreak, source attribution and host transmission studies.

## Methods

### Case history and bacterial culture

The clinical history of the mother-to-child transmission case has been described elsewhere [3]. Briefly, a 7-month old breastfeeding child from a remote region in northern Australia was hospitalized in 2003 with acute cough, fever and tachypnoea. Four days after admission, the mother was observed to have a fever and pleuritic chest pain, and was subsequently diagnosed with mastitis in the left breast. Upon *B. pseudomallei* culture confirmation in the child’s cerebrospinal fluid (CSF), blood, and nasal and throat swabs, the mother was also tested for *B. pseudomallei* infection in blood, sputum and multiple breast milk specimens, and from nasal, throat and rectal swabs. Of these, *B. pseudomallei* was isolated from the mother’s breast milk and sputum (Table 1) [3]. All clinical specimens were cultured on Ashdown’s media as described elsewhere [13]. DNA extractions were performed as previously described [23] on a sweep of the primary culture streak (herein referred to as primary culture sweeps) of each *B. pseudomallei *-positive clinical specimen in an effort to capture potential strain mixtures in these original specimens, and subsequently from individually purified colonies derived from these specimens.

### WGS and *in silico* MLST

As part of the ongoing Darwin Prospective Melioidosis Study (DPMS), which commenced in 1989 [24], all mother and child primary culture sweeps and purified colonies (i.e. isolates) were subjected to WGS using the Illumina HiSeq2500 platform and Illumina Nextera XT chemistry to generate 2×100 bp read data (Australian Genome Research Facility, Melbourne, Australia). WGS was performed on primary culture sweeps and isolates from the mother’s (*n*=6) and child’s (*n*=4) specimens. Reference-assisted draft genome assemblies were performed using mgap v1.0 (default settings) [25], with the closed Australian MSHR1153 genome (CP009271.1 and CP009272.1 for chromosomes 1 and 2, respectively) [26] used as the reference for read mapping and variant calling. We used a reference-assisted assembly approach as, in our experience, doing so results in assemblies with less fragmentation and fewer SNP and small insertion-deletion (indel) errors that require subsequent manual correction. *In silico* MLST was performed by BIGSdb [27], which is embedded within the PubMLST *B. pseudomallei* database available at http://pubmlst.org/bpseudomallei/. For the mixed-strain sample (MSHR1631_Mixed), manual allele assignment was performed by inspecting alignment files using Tablet [28] and parsing SNPs corresponding to the different strain ‘haplotypes’ based on allele abundance.

### Comparative genomics

Comparative genomic analysis was performed with the default settings of SPANDx v3.2 (https://github.com/dsarov/SPANDx) [29], which wraps bwa (Burrows–Wheeler Aligner) [30], SAMtools [31], the Genome Analysis Toolkit (gatk v3.2–2) [32], BEDTools [33] and SNPEff [34] into a single pipeline. Mapping was carried out using the closed Australian genome MSHR1153 [26] as the reference, with the SPANDx *-i* flag enabled to provide indel variant identification. In addition, the SPANDx *-a* and *-v* flags were enabled to permit both the annotation of all SNP and indel variants, and the rapid identification of putative mixtures based on ambiguous (i.e. ‘?’) variant calls in the ‘All_SNPs_annotated.txt’ and ‘All_indels_annotated.txt’ outputs generated by SPANDx.

### Mixture analysis

‘Heterozygous’ SNPs in each isolate were enumerated from the gatk v4.1 HaplotypeCaller [35] VCF output following alignment of reads using bwa [30]. For each heterozygous SNP identified in MSHR1631_Mixed, the depth (i.e. number of reads) supporting each allele was extracted from the VCF file and normalized by the total read depth at each SNP position. One sweep culture, MSHR1631_Mixed, exhibited a substantial number of heterozygous SNPs when compared with all other isolates and sweep cultures, so was further investigated as a possible mixture. Variant identification in MSHR1631_Mixed was determined using gatk v4.1 HaplotypeCaller due to its ability to natively handle polyploid samples. Variant filtering was performed using the parameters described in SPANDx v3.2 [29]. Additionally, to ensure robust variant calling and to assess mixture composition, we tested multiple ploidy settings (*n*=2, 3, 4 and 5).

### Phylogenomic analyses

A maximum parsimony (MP) phylogenetic tree representing a global snapshot of *B. pseudomallei* isolates was reconstructed using orthologous, biallelic, core-genome SNPs identified across 145 publicly available genomes [36] using the default settings of SPANDx and MSHR1153 as the reference genome. The new isolates/sweep cultures sequenced as part of this study were also included in phylogenomic analyses, both with and without the inclusion of MSHR1631_Mixed. The global trees were rooted with MSHR0668 [37], as this strain is the most ancestral *B. pseudomallei* strain according to a large *Burkholderia* spp. phylogeny [15].

To investigate *B. pseudomallei* transmission from mother to child, a combined SNP–indel [11] MP tree containing all available ST-259 isolates was first reconstructed, with the mgap-assembled ST-259 genome MSHR0643 (GenBank ref: VXLH00000000.1) used as the reference for SPANDx analysis. MSHR0643 was chosen as the reference genome as it had the fewest contigs (*n*=93) of any ST-259 strain. Also included in this analysis were seven temporally or geographically distinct ST-259 isolates that were not epidemiologically linked to the mother–baby cases (Table 1). To further investigate putative mutations among mother and baby ST-259 isolates, SPANDx analyses were also performed using the baby’s CSF isolate, MSHR1574, and the mother’s sputum isolate, MSHR1631, as reference genomes, with these comparisons performed using only mother–baby ST-259 isolates. MP phylogenetic tree reconstruction and bootstrapping (300 replicates) were performed using paup* v4.0a165 and visualized with iTOL v4 [38].

### PFGE

PFGE with *Spe*I-digested DNA was performed on mother and child isolates as previously described [39].

## Results and Discussion

**Fig. 1.**
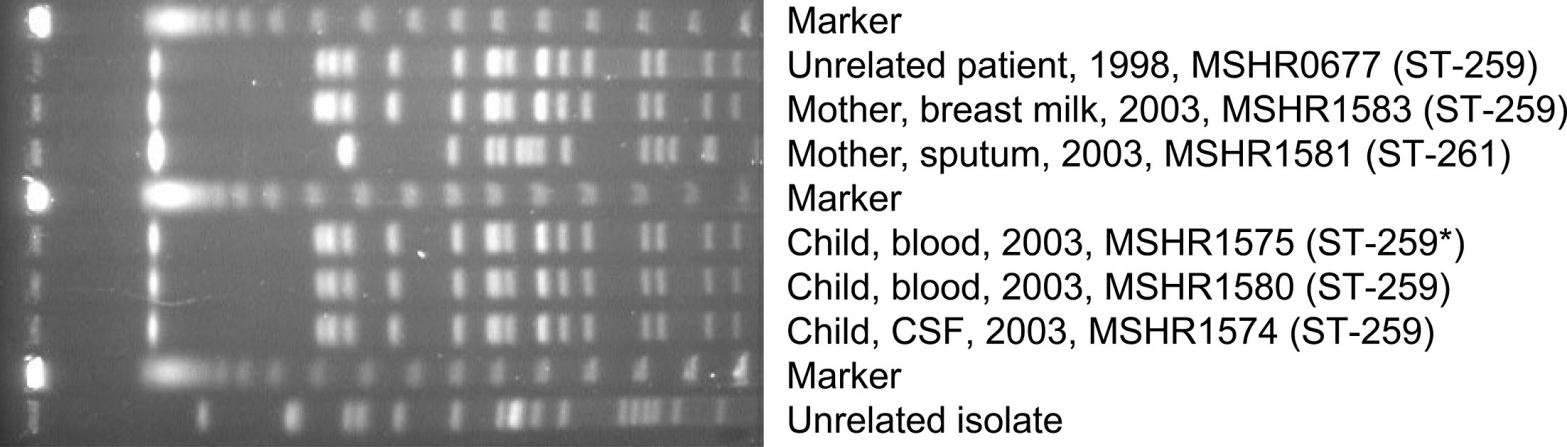
PFGE analysis of mother and child isolates using *Spe*I digestion. *Isolate not subjected to WGS in this study due to culture destruction.

*B. pseudomallei* causes melioidosis, a life-threatening disease with a predicted global incidence of ~165 000 cases annually [2]. Almost all *B. pseudomallei* infections occur via contact with contaminated water or soil, with human-to-human transmission events considered exceedingly rare [40]. Here, we used genomics to examine, in high resolution, one such human-to-human transmission event where a nursing mother with culture-confirmed melioidosis mastitis was suspected to have transmitted *B. pseudomallei* to her child through contaminated breast milk [3]. PFGE analysis on isolates retrieved from the mother and her child shortly after diagnosis identified two pulsotypes in the mother’s sputum isolates (Fig. 1), suggesting a potential polyclonal infection. Consistent with the PFGE findings, *in silico* MLST data showed strains from the mother’s sputum and breast milk matched the CSF- and blood-derived isolates retrieved from the child, with all isolates being ST-259. To further understand this unusual case, WGS was performed on all available specimens from these cases to elucidate transmission dynamics from mother to child, to investigate the potential presence of within-host strain mixtures in the mother and, finally, to examine the effects of strain mixtures on downstream phylogenomic interpretations.

Prior studies have relied upon epidemiological and clinical observations [3, 17, 19, 20], often alongside gel electrophoresis-based genotyping methods [3, 17, 18], to examine cases of suspected *B. pseudomallei* transmission between human hosts. However, these genotyping methods lack the necessary resolution for definitive confirmation of such transmission events, as they only assess a small fraction of the genome. As such, infections arising from independent environmental sources, or even from a single environmental point source as observed in outbreak scenarios [11, 41], cannot be ruled out using such lower-resolution methods.

**Fig. 2.**
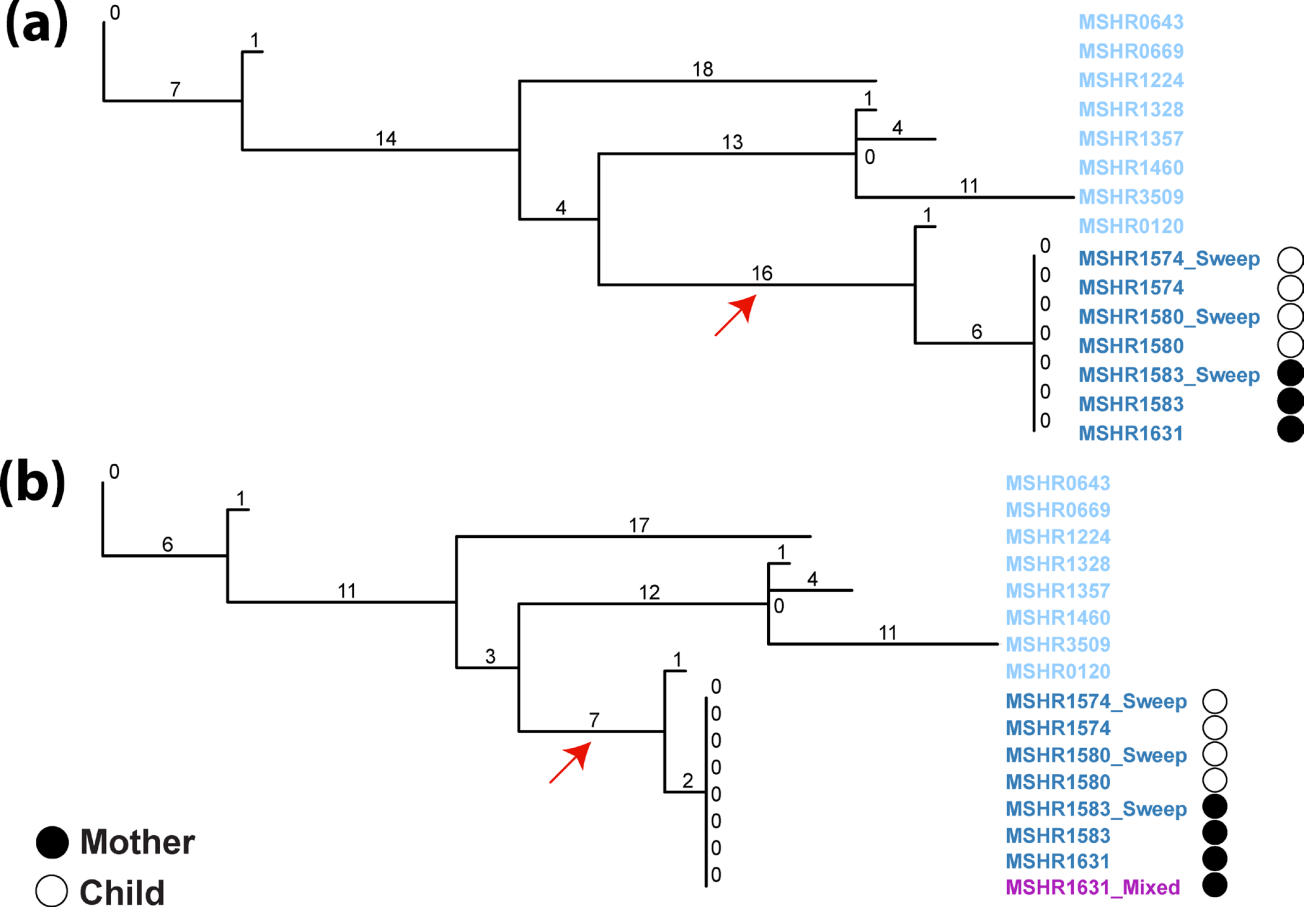
MP phylogenetic analyses of combined SNP–indel characters identified among *B. pseudomallei* ST-259 isolates, including mother and child isolates (dark blue). The MSHR1631_Mixed sample (purple) is a mixture of ST-259 and ST-261 at an 87%:13 % ratio. (a) All ST-259 mother and child isolates were identical, with no observed SNP or indel differences. Mother–child isolates were most closely related to MSHR0120, a clinical ST-259 isolate from the same remote island, which was collected in 1992. (b) The inclusion of a strain mixture (MSHR1631_Mixed; purple) from the mother results in the reduction of informative characters and branch collapse (e.g. red arrows).

To obtain the most epidemiologically robust information from our WGS data, phylogenomic analysis of all mother-child ST-259 isolates was performed using a combined SNP–indel approach, which we have previously shown provides both higher resolution and a better fit with outbreak chronology compared with phylogenomic reconstruction using just SNPs [11]. This approach identified no SNP nor indel differences between the mother and child ST-259 isolates (Fig. 2a). Further comparative genomic analyses examining copy-number variants or larger deletions also failed to find any other genetic variation among the mother–child ST-259 isolates using three different ST-259 reference genomes (MSHR0643 from an unrelated patient 5 years prior to the mother–baby case; the MSHR1631 sputum isolate from the mother; and the MSHR1574 CSF isolate from the baby). Although there will always remain the possibility that the mother and child were infected from a single environmental point source, or that the child infected the mother prior to either becoming symptomatic, our collective clinical, epidemiological and genomic findings point strongly to ST-259 *B. pseudomallei* transmission from mother to child, with breastfeeding being the most likely route of infection. Our findings provide the strongest evidence presented to date that *B. pseudomallei* can transmit between human hosts. This finding raises clinical and biowarfare concerns, particularly in cases where a *B. pseudomallei* strain has developed acquired antimicrobial resistance (AMR) in one human host who subsequently transmits the strain to another. Although acquired AMR in *B. pseudomallei* is relatively uncommon, there are myriad chromosomal mutations that can lead to clinically relevant AMR in *B. pseudomallei* [42], leading to more challenging pathogen eradication [43]. While this phenomenon has not yet been documented, our study demonstrates that human-to-human transfer of an AMR *B. pseudomallei* strain is possible.

To further understand ST-259 diversity on a broader scale, the ST-259 mother–child isolates were compared with seven temporally or geographically distinct clinical ST-259 isolates obtained between 1992 and 2009 from patients living in the Top End region of the Northern Territory, Australia. The mother–child clade was most closely related to MSHR0120, differing by seven variants (Fig. 2). MSHR0120 was retrieved from a patient diagnosed with melioidosis 11 years prior, who lived at the same remote locale as the mother and child. Additionally, minimal differences (between 36 and 45 variants) were observed between the mother–child clade and other ST-259 isolates, suggesting close relatedness of strains within this ST, but a clear difference between the mother–child cases and all other documented ST-259 cases in the Top End region. Taken together, these results provide further evidence for human-to-human *B. pseudomallei* transmission between mother and child.

**Fig. 3.**
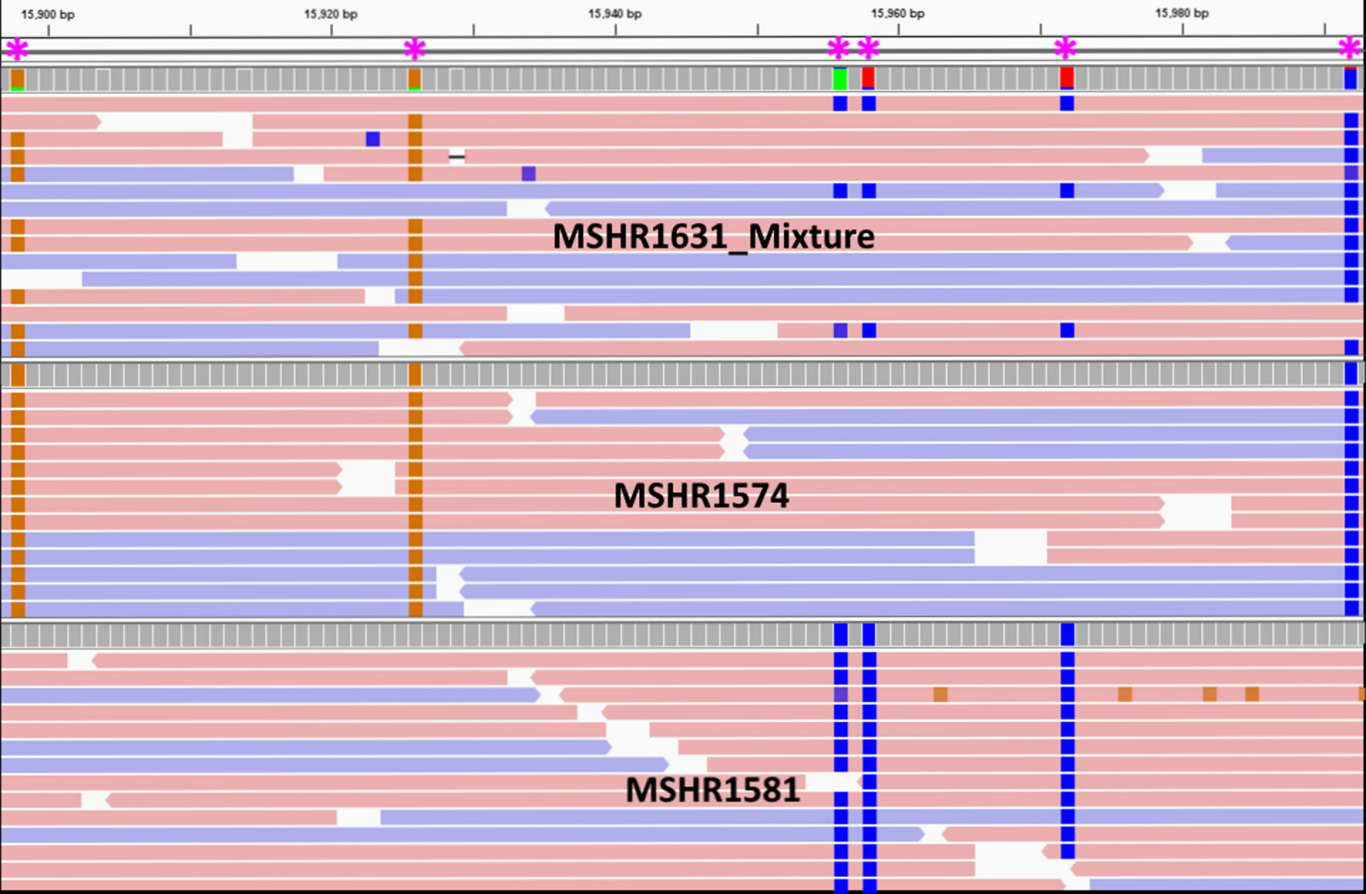
Example of ‘heterozygous’ (i.e. strain mixture) SNP calls at the sequence read level according to gatk HaplotypeCaller. Heterozygous SNP calls in MSHR1631_Mixed (ST-259 and ST-261) were parsed apart by comparing against homozygous SNP calls from MSHR1574 (ST-259) and MSHR1581 (ST-261). Horizontal bars represent forward (red) and reverse (blue) reads aligned against the MSHR1153 reference genome. Coloured boxes represent ‘heterozygous’ SNPs (also indicated by asterisks at the top).

Simultaneous infections with multiple *B. pseudomallei* strains have previously been reported [16, 44, 45]; however, the true rate of polyclonal *B. pseudomallei* infections is unknown. Polyclonality may increase the risk of neurological disease when one or more strains encode a *Burkholderia mallei bimA* (*bimA*_Bm_) genetic variant [46], and may cause issues with accurate point-source attribution in epidemiological investigations if polyclonality is not taken into account. Most clinical microbiological laboratories typically only select a single bacterial pathogen colony for further genotypic and phenotypic characterization, which results in a considerable genetic bottleneck and the loss of strain mixtures from polyclonal clinical specimens. This shortcoming can be overcome using more time-intensive methods, such as the selection of multiple colonies for genetic analysis, sequencing of a ‘sweep’ of primary culture growth for further genetic characterization or by total metagenomic sequencing of the clinical specimen. Due to inherent ethical and technical challenges with metagenomic sequencing of clinical specimens, we chose to genome-sequence culture sweeps and the individual colonies purified from them to identify putative *B. pseudomallei* strain mixtures in the mother and child clinical specimens. Consistent with the PFGE findings, *in silico* MLST and gatk HaplotypeCaller analysis of mother–child sweeps revealed that two distinct strains (ST-259 and ST-261) were found in one of the two sputa retrieved from the mother (MSHR1631_Mixed; Fig. 3), but not in other primary sweep specimens from this patient [1× sputum (MSHR1581_Sweep); 1× breast milk (MSHR1583_Sweep)], nor in the samples obtained from the child [1× CSF (MSHR1574_Sweep); 1× blood (MSHR1580_Sweep)]. WGS of single purified colonies from MSHR1631_Mixed and MSHR1581_Sweep confirmed that both ST-259 and ST-261 were present in this patient’s sputum specimens. Collectively, these results confirm that the mother had a simultaneous infection with two strains, adding to the documented polyclonal *B. pseudomallei* cases.

**Fig. 4.**
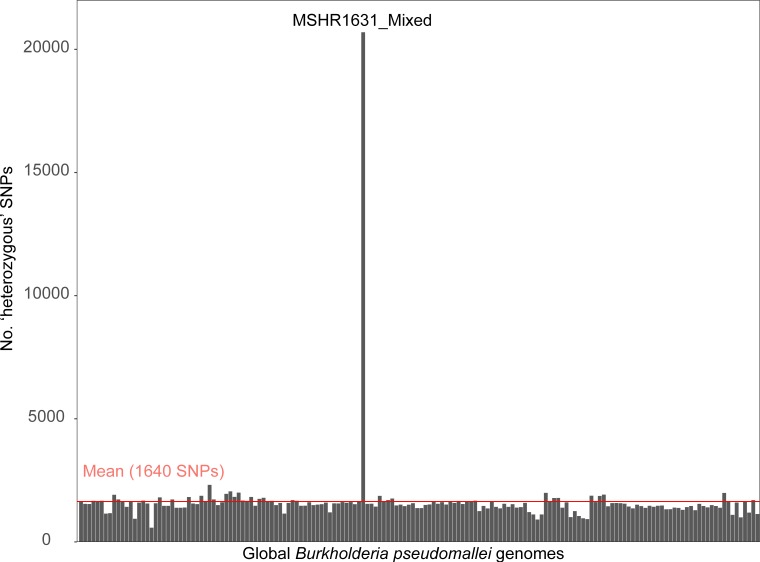
Quantification of ‘heterozygous’ (i.e. strain mixture) SNP calls across all mother–child isolates and a global *B. pseudomallei* genome set. MSHR1631_Mixed contained 12× the mean number of ‘heterozygous’ calls according to the gatk UnifiedGenotyper, indicating the presence of a *B. pseudomallei* strain mixture in this sample. No other analysed genomes contained detectable mixtures.

To better understand this polyclonal infection from a bioinformatic standpoint, we first performed a high-throughput analysis of putative mixtures in our isolate dataset (Table 1) using the SPANDx ‘All_SNPs_annotated.txt’ and ‘All_indels_annotated.txt’ outputs, which incorporates a ‘?’ for ambiguous variant calls according to gatk v3.2–2. This approach readily flagged MSHR1631_MIXED as a probable mixture based on its very high number of ambiguous SNP (18 885 of 41 438 total SNPs; 45.6 %) and indel (1643 of 3328 total indels; 49.4 %) characters compared with all other genomes [SNPs, range 426–518 (1.0–1.3 %); indels, range 165–217 (5.0–6.5 %)]. In other words, MSHR1631_MIXED encoded 40× more ambiguous SNPs and 9× more ambiguous indels than non-mixed strains using this approach. Next, we quantified the number of high-quality heterozygous SNPs in MSHR1631_Mixed using gatk v4.1. Haploid genomes such as bacterial genomes do not encode heterozygous SNPs; therefore, heterozygous SNPs are typically ignored by bacterial genome variant-calling software. The inclusion of heterozygous SNPs in an analysis of the mother–child isolates amongst a global dataset of *B. pseudomallei* genomes showed that MSHR1631_Mixed contained 12× the mean number of heterozygous SNPs compared with all other mother–child samples (Fig. 4). In total, 34 567 SNPs were identified in this sample, 47.8 % of which were ‘heterozygous’. In contrast, a mean of 29 914 SNPs were identified in the other nine mother–baby samples, of which only 5.2 % were ‘heterozygous’. Next, homozygous SNPs identified in representative pure isolates (MSHR1574 for ST-259; MSHR1581 for ST-261) were used to identify the strain origin of each heterozygous allele from MSHR1631_Mixed SNPs. Using this method, 96 % of heterozygous SNPs were matched to the correct strain. ST-259 was the dominant clone (averaging 87 % of reads at each variant) and ST-261 was present as a minor allelic component (averaging 13 % of reads at each variant). No evidence of a tertiary strain was observed in MSHR1631_Mixed when different ploidy settings were tested, indicating that no other strains were present.

The utility of SNP data derived from WGS to identify and study mixtures has been demonstrated in different diploid and polyploid organisms [47–49]. Current approaches for identifying mixtures in bacterial organisms include proportion estimates against a database of known STs or species-specific marker genes [50, 51], which requires prior knowledge of the specific bacterial population, or long-read sequencing [52], which is currently costly and error-prone when used in isolation. Other studies have identified mixtures in bacterial genomes using the UnifiedGenotyper function of gatk v3 to detect ambiguous variants in a similar fashion to our SPANDx analysis [53], or genotype binning based on differences in haplotype depth of coverage in five cases of mixed *Mycobacterium tuberculosis* infections, including one case where three genotypes were suspected in a single patient [54]. Bioinformatic solutions are available for ploidy inference of eukaryotic organisms [47–49, 55], which rely on the depth ratio of the two most abundant alleles sequenced for all heterozygous SNP positions across the genome (also referred to as ‘allele balance’). Such approaches assume SNP allele balances remain relative to each other; for example in a diploid sample, 50 % of reads would support one allele and the other 50 % would support the other allele [47]. However, the allele balance assumption does not hold in bacterial mixtures, which may contain mixed ratios of any proportion. Despite this shortcoming, we demonstrated the feasibility of using SNP and read depth data to parse apart bacterial mixtures without any prior knowledge of the mixture composition. This approach relies on sequencing at a depth of ≥50× to ensure adequate sampling of a minor allelic component present at a~5–10 % proportion [42]. However, in our view, such an approach is only suited for parsing apart mixtures of two strains. Although the major strain is potentially identifiable in ≥3-strain mixtures, parsing apart minor components is a complex problem that remains largely unresolved using short-read data.

**Fig. 5.**
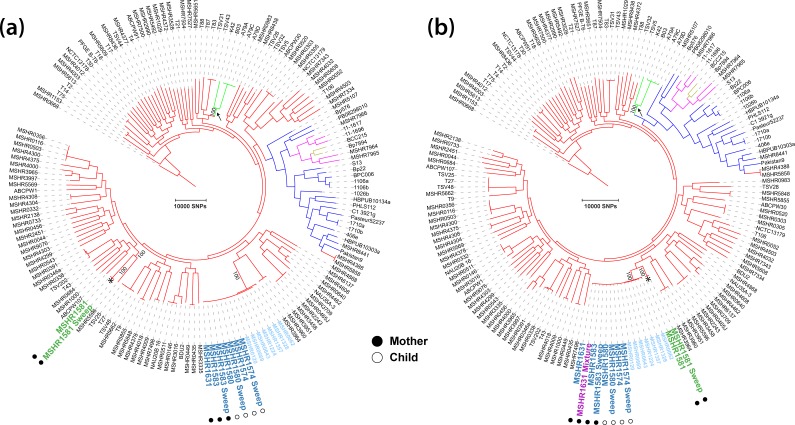
Global MP phylogenetic analyses demonstrating the effects of strain mixtures on tree topology. Branch colours denote geographical origin of *B. pseudomallei* strains: red, Australian isolates; blue, Asian isolates; pink, African isolates; lime green, Papua New Guinean isolates; gold, South American isolates. Isolate names in dark blue text, ST-259 mother–child isolates; light blue text, temporally or geographically distinct ST-259 isolates; green text, ST-261 mother isolates; purple text, MSHR1631_Mixed sample. (a) Exclusion of the mixed genome, MSHR1631_Mixed, results in correct topology and separation of ST-259 and ST-261 according to previous global *B. pseudomallei* phylogenies [13, 15, 36]; these two STs differ by >20 000 SNPs. (b) Inclusion of MSHR1631_Mixed greatly alters topology, leading to incorrect isolate and clade clustering, and collapsed branches in the clade containing MSHR1631_Mixed. Specifically, the ST-261 isolates (indicated by asterisks) cluster incorrectly with ST-259, with branch collapse observed in this clade. The Papua New Guinean isolates are also incorrectly placed in this phylogeny (indicated by black arrows). The number of characters used to reconstruct each tree differs by 14 503 SNPs (a, 207 209 SNPs; b, 192 706 SNPs), resulting in branch collapse.

Finally, we investigated the effects of strain mixtures on phylogenomic reconstruction to determine whether the inclusion of even one mixture had undesirable effects on tree topology and phylogenetic inference. Phylogenomic analyses were performed with the ST-259 (Fig. 2) and global (Fig. 5) datasets, both with (Figs 2b and 5b) and without (Figs 2a and 5a) MSHR1631_Mixed inclusion. Tree comparisons identified two confounding issues in the trees containing MSHR1631_Mixed: phylogenetic incongruence [56] in the global dataset, which resulted in multiple instances of incorrect clade placement, and branch collapse in both the ST-259 and global datasets, which was caused by the removal of informative characters for phylogenetic reconstruction due to the presence of ambiguous characters in MSHR1631_Mixed. In the ST-259 tree, the number of SNP–indel characters separating isolates decreased from 35 to 21 variants (Fig. 2b). In turn, the inferred relatedness between the mother–child ST-259 isolates and other ST-259 isolates was exaggerated due to this branch collapse (Fig. 2b; red arrow). In the global dataset, branch collapse reduced the total number of informative characters available for tree reconstruction by 18 051 SNPs when compared with the non-mixed phylogeny. Phylogenetic incongruence was also evident in the global tree, whereby ST-261 isolates (MSHR1581_Sweep and MSHR1581; green text) incorrectly resided in the same clade as ST-259 (Fig. 5b; asterisk). In contrast, the non-mixed dataset separated these two STs by approximately 20 000 SNPs, with clear separation of these clades (Fig. 5a). Surprisingly, branches across both trees had very high bootstrap support values at the ST-261 and ST-259 clades despite branch collapse and phylogenetic incongruence in the mixed dataset. Of further concern, the phylogeny containing MSHR1631_Mixed caused incorrect geographical assignment of the Papua New Guinean clade, unexpectedly shifting its known grouping with Australian strains [15, 57] to the Asian clade; this incorrect placement received very high bootstrap support (Fig. 5b).

The negative effects of strain mixtures on phylogenomic inference highlights the importance of strict quality controls throughout each stage of the experiment, especially during computational analysis. Bioinformatically, bacterial mixtures can be readily detected, as demonstrated in this study. However, standard practice in microbial variant calling pipelines is to report only homozygous variants for downstream analysis, with heterozygous SNPs typically ignored. Additionally, most phylogenetic reconstruction software treat heterozygous SNPs as missing or non-informative characters, even when encoded with IUPAC-ambiguous characters [58]. Our results provide unequivocal evidence that caution is needed in phylogenomic interpretation when dealing with potential strain mixtures. As these mixtures are not easily identifiable from phylogenetic analysis, it is prudent that all microbial genomics studies include a mixture screening assessment of all genomes prior to variant calling and phylogenomic reconstruction to avoid removing phylogenetic informative characters, which we show can readily result in branch collapse and phylogenetic incongruence. Based on our findings, it is our strong recommendation that any sample demonstrating evidence of a mixture should be entirely omitted from a dataset, particularly if the purpose is to examine fine-scale differences between or among closely related samples, such as in the current study. This criterion may be relaxed if a given study’s aims can tolerate branch collapse or phylogenetic incongruence; however, the effects of both mixture inclusion and exclusion should be investigated in such cases to understand the potentially confounding impact of the mixture(s) on the dataset under investigation. In such instances where a mixed genome is essential for a study, additional laboratory passage to purify the mixture(s) should be considered. In cases where this is not possible, longer-read sequencing may assist with parsing apart allele mixtures.

In conclusion, we demonstrate the utility of comparative genomics to both confirm human-to-human *B. pseudomallei* transmission and to identify simultaneous infection with multiple *B. pseudomallei* strains. Using a naturally occurring mixed genome comprising two strains at an 87%:13 % ratio, we describe an effective method to accurately identify and quantify such mixtures from WGS data, and highlight the confounding effects that even a single mixed genome can place on accurate phylogenomic interpretations for both closely related (e.g. single ST) and species-wide phylogenies. Our findings demonstrate the essentiality of assessing all microbial genome datasets for the presence of strain mixtures as a routine part of sequence data quality control. We strongly recommend that such mixtures be removed prior to phylogenomic analysis to avoid erroneous misinterpretations of strain relatedness.

## Data bibliography

Aziz, A. Accession numbers and references retrieved from the following paper – Sarovich DS, Garin B, De Smet B, Kaestli M, Mayo M *et al*. Phylogenomic analysis reveals an Asian origin for African *Burkholderia pseudomallei* and further supports melioidosis endemicity in Africa. *mSphere* 2016;1:e00089-15 – for the 145 global *Burkholderia pseudomallei* isolate dataset, are available on Figshare: https://doi.org/10.6084/m9.figshare.9840212 (2019).

## References

[1] Limmathurotsakul D, Peacock SJ (2011). Melioidosis: a clinical overview. Br Med Bull.

[2] Limmathurotsakul D, Golding N, Dance DAB, Messina JP, Pigott DM (2016). Predicted global distribution of *Burkholderia pseudomallei* and burden of melioidosis. Nat Microbiol.

[3] Ralph A, McBride J, Currie BJ (2004). Transmission of *Burkholderia pseudomallei* via breast milk in northern Australia. Pediatr Infect Dis J.

[4] Limmathurotsakul D, Wongratanacheewin S, Day NPJ, Teerawattanasook N, Chaowagul W (2010). Increasing incidence of human melioidosis in northeast Thailand. Am J Trop Med Hyg.

[5] Schweizer HP (2012). Mechanisms of antibiotic resistance in *Burkholderia pseudomallei*: implications for treatment of melioidosis. Future Microbiol.

[6] Wiersinga WJ, Currie BJ, Peacock SJ (2012). Melioidosis. N Engl J Med.

[7] Butler D (2012). Viral research faces clampdown. Nature.

[8] Aziz A, Sarovich DS, Harris TM, Kaestli M, McRobb E (2017). Suspected cases of intracontinental *Burkholderia pseudomallei* sequence type homoplasy resolved using whole-genome sequencing. Microb Genom.

[9] Dale J, Price EP, Hornstra H, Busch JD, Mayo M (2011). Epidemiological tracking and population assignment of the non-clonal bacterium, *Burkholderia pseudomallei*. PLoS Negl Trop Dis.

[10] Pearson T, Giffard P, Beckstrom-Sternberg S, Auerbach R, Hornstra H (2009). Phylogeographic reconstruction of a bacterial species with high levels of lateral gene transfer. BMC Biol.

[11] McRobb E, Sarovich DS, Price EP, Kaestli M, Mayo M (2015). Tracing melioidosis back to the source: using whole-genome sequencing to investigate an outbreak originating from a contaminated domestic water supply. J Clin Microbiol.

[12] Engelthaler DM, Bowers J, Schupp JA, Pearson T, Ginther J (2011). Molecular investigations of a locally acquired case of melioidosis in southern AZ, USA. PLoS Negl Trop Dis.

[13] Price EP, Sarovich DS, Smith EJ, MacHunter B, Harrington G (2016). Unprecedented melioidosis cases in northern Australia caused by an Asian *Burkholderia pseudomallei* strain identified by using large-scale comparative genomics. Appl Environ Microbiol.

[14] McCombie RL, Finkelstein RA, Woods DE (2006). Multilocus sequence typing of historical *Burkholderia pseudomallei* isolates collected in Southeast Asia from 1964 to 1967 provides insight into the epidemiology of melioidosis. J Clin Microbiol.

[15] Price EP, Currie BJ, Sarovich DS (2017). Genomic insights into the melioidosis pathogen, *Burkholderia pseudomallei*. Curr Trop Med Rep.

[16] Price EP, Sarovich DS, Viberg L, Mayo M, Kaestli M (2015). Whole-genome sequencing of *Burkholderia pseudomallei* isolates from an unusual melioidosis case identifies a polyclonal infection with the same multilocus sequence type. J Clin Microbiol.

[17] Holland DJ, Wesley A, Drinkovic D, Currie BJ (2002). Cystic fibrosis and *Burkholderia pseudomallei* infection: an emerging problem?. Clin Infect Dis.

[18] Kunakorn M, Jayanetra P, Tanphaichitra D (1991). Man-to-man transmission of melioidosis. The Lancet.

[19] McCormick JB, Sexton DJ, McMurray JG, Carey E, Hayes P (1975). Human-to-human transmission of *Pseudomonas pseudomallei*. Ann Intern Med.

[20] Abbink FC, Orendi JM, de Beaufort AJ (2001). Mother-to-child transmission of *Burkholderia pseudomallei*. N Engl J Med.

[21] Lumbiganon P, Pengsaa K, Puapermpoonsiri S, Puapairoj A (1988). Neonatal melioidosis: a report of 5 cases. Pediatr Infect Dis J.

[22] Choy JL, Mayo M, Janmaat A, Currie BJ (2000). Animal melioidosis in Australia. Acta Trop.

[23] Currie BJ, Gal D, Mayo M, Ward L, Godoy D (2007). Using BOX-PCR to exclude a clonal outbreak of melioidosis. BMC Infect Dis.

[24] Currie BJ, Ward L, Cheng AC (2010). The epidemiology and clinical spectrum of melioidosis: 540 cases from the 20 year Darwin prospective study. PLoS Negl Trop Dis.

[25] Sarovich D (2017).

[26] Johnson SL, Baker AL, Chain PS, Currie BJ, Daligault HE (2015). Whole-Genome sequences of 80 environmental and clinical isolates of *Burkholderia pseudomallei*. Genome Announc.

[27] Jolley KA, Bray JE, Maiden MCJ (2018). Open-access bacterial population genomics: BIGSdb software, the PubMLST.org website and their applications. Wellcome Open Res.

[28] Milne I, Stephen G, Bayer M, Cock PJA, Pritchard L (2013). Using tablet for visual exploration of second-generation sequencing data. Brief Bioinform.

[29] Sarovich DS, Price EP (2014). SPANDx: a genomics pipeline for comparative analysis of large haploid whole genome re-sequencing datasets. BMC Res Notes.

[30] Li H (2013). Aligning sequence reads, clone sequences and assembly contigs with BWA-MEM. ArXiv.

[31] Li H, Handsaker B, Wysoker A, Fennell T, Ruan J (2009). The Sequence Alignment/Map format and SAMtools. Bioinformatics.

[32] McKenna A, Hanna M, Banks E, Sivachenko A, Cibulskis K (2010). The genome analysis toolkit: a MapReduce framework for analyzing next-generation DNA sequencing data. Genome Res.

[33] Quinlan AR, Hall IM (2010). BEDTools: a flexible suite of utilities for comparing genomic features. Bioinformatics.

[34] Cingolani P, Platts A, Wang LL, Coon M, Nguyen T (2012). A program for annotating and predicting the effects of single nucleotide polymorphisms, SnpEff: SNPs in the genome of *Drosophila melanogaster* strain w1118; iso-2; iso-3. Fly.

[35] Van der Auwera GA, Carneiro MO, Hartl C, Poplin R, Del Angel G (2013). From FastQ data to high confidence variant calls: the genome analysis toolkit best practices pipeline. Curr Protoc Bioinformatics.

[36] Sarovich DS, Garin B, De Smet B, Kaestli M, Mayo M (2016). Phylogenomic analysis reveals an Asian origin for African *Burkholderia pseudomallei* and further supports melioidosis endemicity in Africa. mSphere.

[37] Johnson SL, Bishop-Lilly KA, Ladner JT, Daligault HE, Davenport KW (2015). Complete genome sequences for 59 *Burkholderia* isolates, both pathogenic and near neighbor. Genome Announc.

[38] Letunic I, Bork P (2019). Interactive tree of life (iTOL) v4: recent updates and new developments. Nucleic Acids Res.

[39] Gal D, Mayo M, Smith-Vaughan H, Dasari P, McKinnon M (2004). Contamination of hand wash detergent linked to occupationally acquired melioidosis. Am J Trop Med Hyg.

[40] Wiersinga WJ, Virk HS, Torres AG, Currie BJ, Peacock SJ (2018). Melioidosis. Nat Rev Dis Primers.

[41] Inglis TJ, Garrow SC, Adams C, Henderson M, Mayo M (1999). Acute melioidosis outbreak in Western Australia. Epidemiol Infect.

[42] Madden DE, Webb JR, Steinig EJ, Mayo M, Currie BJ Taking the next-gen step: comprehensive antibiotic resistance detection from *Burkholderia pseudomallei* genomes. BioRxiv.

[43] Sarovich DS, Webb JR, Pitman MC, Viberg LT, Mayo M (2018). Raising the stakes: loss of efflux pump regulation decreases meropenem susceptibility in *Burkholderia pseudomallei*. Clin Infect Dis.

[44] Pitt TL, Trakulsomboon S, Dance DAB (2007). Recurrent melioidosis: possible role of infection with multiple strains of *Burkholderia pseudomallei*. J Clin Microbiol.

[45] Limmathurotsakul D, Wuthiekanun V, Chantratita N, Wongsuvan G, Thanwisai A (2007). Simultaneous infection with more than one strain of *Burkholderia pseudomallei* is uncommon in human melioidosis. J Clin Microbiol.

[46] Sarovich DS, Price EP, Webb JR, Ward LM, Voutsinos MY (2014). Variable virulence factors in *Burkholderia pseudomallei* (melioidosis) associated with human disease. PLoS One.

[47] Augusto Corrêa Dos Santos R, Goldman GH, Riaño-Pachón DM (2017). ploidyNGS: visually exploring ploidy with next generation sequencing data. Bioinformatics.

[48] Churchill JD, Stoljarova M, King JL, Budowle B (2018). Massively parallel sequencing-enabled mixture analysis of mitochondrial DNA samples. Int J Legal Med.

[49] Weiß CL, Pais M, Cano LM, Kamoun S, Burbano HA (2018). nQuire: a statistical framework for ploidy estimation using next generation sequencing. BMC Bioinformatics.

[50] Truong DT, Tett A, Pasolli E, Huttenhower C, Segata N (2017). Microbial strain-level population structure and genetic diversity from metagenomes. Genome Res.

[51] Eyre DW, Cule ML, Griffiths D, Crook DW, Peto TEA (2013). Detection of mixed infection from bacterial whole genome sequence data allows assessment of its role in *Clostridium difficile* transmission. PLoS Comput Biol.

[52] Driscoll CB, Otten TG, Brown NM, Dreher TW (2017). Towards long-read metagenomics: complete assembly of three novel genomes from bacteria dependent on a diazotrophic cyanobacterium in a freshwater lake co-culture. Stand Genomic Sci.

[53] Bos KI, Harkins KM, Herbig A, Coscolla M, Weber N (2014). Pre-Columbian mycobacterial genomes reveal seals as a source of new world human tuberculosis. Nature.

[54] Kay GL, Sergeant MJ, Zhou Z, Chan JZ-M, Millard A (2015). Eighteenth-century genomes show that mixed infections were common at time of peak tuberculosis in Europe. Nat Commun.

[55] Knaus BJ, Grünwald NJ (2017). vcfr: a package to manipulate and visualize variant call format data in R. Mol Ecol Resour.

[56] Philippe H, Brinkmann H, Lavrov DV, Littlewood DTJ, Manuel M (2011). Resolving difficult phylogenetic questions: why more sequences are not enough. PLoS Biol.

[57] Baker AL, Pearson T, Sahl JW, Hepp C, Price EP (2018). *Burkholderia pseudomallei* distribution in Australasia is linked to paleogeographic and anthropogenic history. PLoS One.

[58] Kates HR, Johnson MG, Gardner EM, Zerega NJC, Wickett NJ (2018). Allele phasing has minimal impact on phylogenetic reconstruction from targeted nuclear gene sequences in a case study of *Artocarpus*. Am J Bot.

